# Case report: Ectopic thyroid tissue found in a liver with hepatocellular carcinoma

**DOI:** 10.3389/fsurg.2022.963182

**Published:** 2022-09-23

**Authors:** Zhanbo Wang, Jing Yuan, Jie Li

**Affiliations:** Department of Pathology, The First Medical Center, Chinese PLA General Hospital, Beijing, China

**Keywords:** ectopic thyroid tissue, hepatocellular carcinoma, thyroid transcription factor-1, glypican3, thyroid follicular carcinoma

## Abstract

**Background:**

Concomitant intrahepatic ectopic thyroid is rare in patients with hepatocellular carcinoma. Thyroid follicular structures outside the hepatocellular carcinoma lesions are regarded as satellite nodules or intrahepatic metastases of hepatocellular carcinoma, which often leads to misdiagnosis and overtreatment of hepatocellular carcinoma patients.

**Case presentation:**

We report the case of an 83-year-old man with moderately differentiated hepatocellular carcinoma (2.5 cm) whose liver contained ectopic thyroid tissue. An encapsulated, multinodular grayish-yellow mass and multiple satellite nodules were detected and removed by right hepatic lobectomy. Microscopically, the main tumor displayed a predominant trabecular, cord-like structure. Liver tissue 0.5 cm from the tumor had a benign-appearing follicular thyroid structure. The follicles contained colloid tissue and were lined with low cuboidal cells with scant cytoplasm; lymphatic tissue was also present in the area. The hepatocellular carcinoma cells were positive for hepatocyte antigen and glypican-3 and negative for cytokeratin 19. The follicular thyroid cells expressed thyroglobulin, PAX8, and thyroid transcription factor-1. A metastatic thyroid neoplasm was excluded clinically and by ultrasound and computed tomography. One month after surgery, all of the patient's serological markers were normal; no tumor recurrence or metastasis has been detected for 7 postoperative months.

**Conclusions:**

The finding of ectopic thyroid tissue in the liver of a patient with hepatocellular carcinoma is very rare. The possibility of hepatocellular carcinoma forming satellite nodules and intrahepatic metastasis should be ruled out first and immunohistochemistry may be definitive in making the diagnosis. Further examination is needed to exclude thyroid cancer liver metastases.

## Introduction

Malignant hepatic tumors, including hepatocellular carcinoma, cholangiocarcinoma, and neuroendocrine tumors, may have a follicular thyroid structure (1, 2); ectopic thyroid tissue is rarely reported (3). Ectopic tissue in the liver may derive from structures such as the adrenal gland, pancreas, and spleen (4–6). When it is present, the possibility of thyroid cancer metastasis must be excluded by studying the patient's medical history and conducting ultrasound examination. No case of ectopic thyroid tissue in the liver of a patient with hepatocellular carcinoma has been described in the literature. Here, we present a case of this rare condition.

## Case presentation

**Figure 1:**
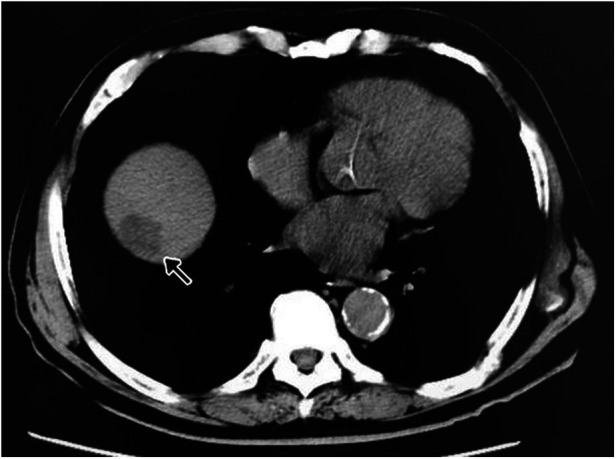
Ct image with irregular and slightly low-density shadows indicating a hepatocellular carcinoma lesion in the right lobe of the liver, near the top of the diaphragm (arrow).

An 83-year-old man presented with a 6-month history of dull pain in the right abdomen. Serological examination revealed that he was negative for hepatitis B surface antigen and hepatitis C virus antibodies. On computed tomography examination, irregular and slightly low-density (∼26 HU) shadows were observed in the right lobe of the liver, near the top of the diaphragm, representing a tumor measuring 29 × 23 mm with clear boundaries (Figure 1). Serological tests revealed that the patient had a carcinoembryonic antigen level of 5.38 µg/L (normal range, 0–5.0 µg/L), alpha fetoprotein level of 3.36 µg/L (normal range, 0–20 µg/L), carbohydrate antigen 19–9 level of 36.97 µ/ml (normal range, 0–37 µ/ml), and cancer antigen-125 level of 11.42 µ/ml (normal range, 0–35 µ/ml).

**Figure 2:**
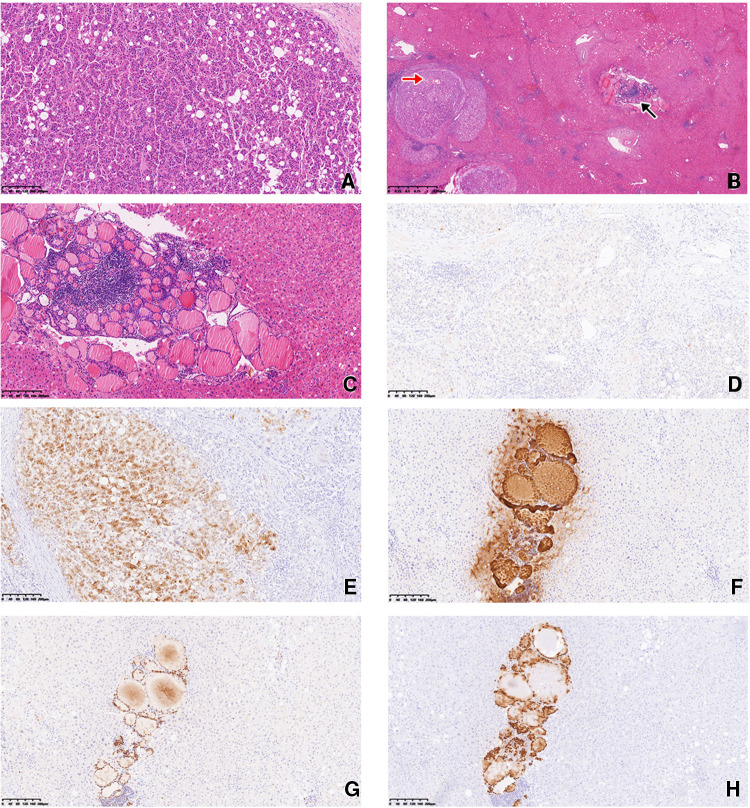
(**A**) The hepatocellular carcinoma had a trabecular structure, with some translucent cells. H/E staining, magnification 100×. (**B**) Multiple satellite nodules were present around the main tumor nodule (red arrow). Liver tissue 0.5 cm from the main tumor had a benign-appearing follicular thyroid structure (black arrow). H/E staining, magnification 20×. (**C**) The follicular epithelial cells showed no atypia. H/E staining, magnification 100×. (**D**) Immunohistochemical staining of the hepatocellular carcinoma was negative for CK-19 (**D**) and positive for GPC-3 (**E**). Magnification 100×. Immunohistochemical staining of the ectopic thyroid epithelial cells was positive for TG (**F**), PAX-8 (**G**), and TTF-1 (**H**). Magnification 100×.

During the operation, the tumor was found to be located in the eighth segment of the right lobe of the liver; thus, the right hepatic lobe was partially resected. The tumor was a grayish-yellow mass (2.5 × 2 × 2 cm) located under the liver capsule with a clear boundary from surrounding tissues. Microscopic observation revealed that it was moderately differentiated hepatocellular carcinoma with a cord-like, trabecular structure and a pattern of local pseudoglandular growth. Some tumor cells were translucent, and multiple satellite nodules were found around the main tumor nodule (Figures 2A,B). An area of liver tissue (∼0.3 cm) located 0.5 cm from the main tumor had a benign-appearing follicular thyroid structure (Figures 2B,C). The lesion was too small to appear on CT images. The follicles contained colloid tissue and were lined with low cuboidal cells with scant cytoplasm; lymphatic tissue was also present in the area.

Immunohistochemical (IHC) analysis of the hepatocellular carcinoma revealed hepatocyte antigen positivity, cytokeratin-19 negativity (Figure 2D), and glypican-3 (GPC-3) positivity (Figure 2E). Epithelial cells in the area with a follicular thyroid structure showed no atypia; they were negative for hepatocyte paraffin 1 (Hep Par-1) and positive for thyroglobulin (TG) (Figure 2F), PAX8 (Figure 2G), and thyroid transcription factor-1 (TTF-1; Figure 2H).

The patient had no history of a thyroid tumor, and thyroid function test results were normal. Thyroid hormone levels were in normal ranges (triiodothyronine, 1.27 ng/mL; thyroxine, 10.5 g/dl; free triiodothyronine, 3.84 pmol/L; and free thyroxine, 17.76 pmol/L).

Ultrasound examination showed no primary thyroid tumor. The final diagnosis was moderately differentiated hepatocellular carcinoma with heterotopic thyroid tissue in the liver. One month after surgery, all of the patient's serological markers were normal; no tumor recurrence or metastasis has been detected for 7 postoperative months.

## Discussion and conclusions

Ectopic tissue in the liver is occasionally reported; adrenal tissues and tumors of adrenal origin (i.e., adrenal heterotopia) are relatively common, found in 9.9% of unselected autopsy cases in one study (7). A search of the PubMed database yielded no report of a hepatocellular carcinoma case in which ectopic thyroid tissue was found in the liver, as in the case reported here.

Ectopic thyroid tissue is the result of abnormal embryonic development of the thyroid. Normally, the primordial thyroid base descends to a location between the thyroid cartilage and sixth tracheal cartilage ring before birth, where it develops into a normal thyroid. When this process fails, the thyroid base may remain between the foramen cecum of the tongue and the thyroid isthmus. Heterotopia of the thyroid gland is a relatively rare condition that primarily involves the liver.

The presence of ectopic thyroid tissue has no specific clinical manifestation, and preoperative diagnosis is difficult. Depending on the anatomical position of this tissue, patients may have non-specific clinical symptoms, such as wheezing and dyspnea, which are easily misdiagnosed and mistreated. The patient described here had no related symptom, and the ectopic thyroid lesion was too small to be detected by imaging.

Hepatic ectopic thyroid tissue must be differentiated from hepatic tumors and pseudolymphoma. Metastasis of a malignant thyroid tumor must be ruled out; distant metastases of thyroid cancer usually involve the lung, bone, and brain, with uncommon sites including the kidney, liver, pancreas, adrenal gland, and ovary (8). Follicular thyroid carcinoma may be difficult to differentiate from ectopic thyroid follicles; ultrasound of the thyroid gland and the patient's medical history are crucial for such differentiation. In the present case, the patient had no thyroid tumor or history thereof, and showed normal thyroid function. The structure of the ectopic thyroid follicles was normal, and the follicular epithelial cells showed no atypia. Thus, thyroid cancer metastasis could be excluded.

When ectopic thyroid tissue with a follicular structure is detected around a hepatocellular carcinoma lesion, as in the present case, the possibility of hepatic epithelial cancer with a thyroid follicular structure must be ruled out (9). Hepatocellular carcinoma that mimics the follicular thyroid structure, especially with a pseudoglandular growth pattern and bile production, is not uncommon. Intrahepatic cholangiocarcinoma and neuroendocrine tumors that mimic the thyroid structure have also been reported (1, 2). In such cases, a single IHC marker cannot be relied on; panels including Hep Par-1, GPC-3, TTF-1, PAX8, TG, synaptophysin, chromogranin A, CD56, and other indicators aid the diagnosis. In the present case, the follicular epithelium was TTF-1, PAX8, and TG positive, indicating that a malignant hepatic epithelial tumor imitating the follicular thyroid structure could be ruled out.

In conclusion, the finding of ectopic thyroid tissue in the liver of a patient with hepatocellular carcinoma is very rare. In such cases, primary tumors of the liver with follicular thyroid morphology, including hepatocellular carcinoma, intrahepatic cholangiocarcinoma, and neuroendocrine tumors, should first be ruled out. Especially in patients with primary liver malignancies, ectopic thyroid lesions may be misdiagnosed as satellite tumor nodules, which may lead to misjudgment of the patient's prognosis and over-treatment. It is also important to consider the patient's history thoroughly and to use imaging to exclude thyroid cancer metastasis.

## Data Availability

The raw data supporting the conclusions of this article will be made available by the authors, without undue reservation.
